# KDM1A regulates nephron progenitor programs and is associated with human kidney disease risk

**DOI:** 10.1093/ndt/gfag073

**Published:** 2026-05-01

**Authors:** Lakshmi P Kolligundla, Dhanunjay Mukhi, Jonathan Levinsohn, Andi Bergerson, Yanjuan Hou, Hongbo Liu, Brain C Capell, Katalin Susztak

**Affiliations:** Renal, Electrolyte, and Hypertension Division, Department of Medicine, Perelman School of Medicine, University of Pennsylvania, Philadelphia, PA, USA; Institute for Diabetes, Obesity, and Metabolism, Perelman School of Medicine, University of Pennsylvania, Philadelphia, PA, USA; Penn/CHOP Kidney Innovation Center, Perelman School of Medicine, University of Pennsylvania, Philadelphia, PA, USA; Department of Genetics, Perelman School of Medicine, University of Pennsylvania, Philadelphia, PA, USA; Renal, Electrolyte, and Hypertension Division, Department of Medicine, Perelman School of Medicine, University of Pennsylvania, Philadelphia, PA, USA; Institute for Diabetes, Obesity, and Metabolism, Perelman School of Medicine, University of Pennsylvania, Philadelphia, PA, USA; Penn/CHOP Kidney Innovation Center, Perelman School of Medicine, University of Pennsylvania, Philadelphia, PA, USA; Department of Genetics, Perelman School of Medicine, University of Pennsylvania, Philadelphia, PA, USA; Renal, Electrolyte, and Hypertension Division, Department of Medicine, Perelman School of Medicine, University of Pennsylvania, Philadelphia, PA, USA; Institute for Diabetes, Obesity, and Metabolism, Perelman School of Medicine, University of Pennsylvania, Philadelphia, PA, USA; Penn/CHOP Kidney Innovation Center, Perelman School of Medicine, University of Pennsylvania, Philadelphia, PA, USA; Department of Genetics, Perelman School of Medicine, University of Pennsylvania, Philadelphia, PA, USA; Renal, Electrolyte, and Hypertension Division, Department of Medicine, Perelman School of Medicine, University of Pennsylvania, Philadelphia, PA, USA; Institute for Diabetes, Obesity, and Metabolism, Perelman School of Medicine, University of Pennsylvania, Philadelphia, PA, USA; Penn/CHOP Kidney Innovation Center, Perelman School of Medicine, University of Pennsylvania, Philadelphia, PA, USA; Department of Genetics, Perelman School of Medicine, University of Pennsylvania, Philadelphia, PA, USA; Renal, Electrolyte, and Hypertension Division, Department of Medicine, Perelman School of Medicine, University of Pennsylvania, Philadelphia, PA, USA; Institute for Diabetes, Obesity, and Metabolism, Perelman School of Medicine, University of Pennsylvania, Philadelphia, PA, USA; Penn/CHOP Kidney Innovation Center, Perelman School of Medicine, University of Pennsylvania, Philadelphia, PA, USA; Department of Genetics, Perelman School of Medicine, University of Pennsylvania, Philadelphia, PA, USA; Renal, Electrolyte, and Hypertension Division, Department of Medicine, Perelman School of Medicine, University of Pennsylvania, Philadelphia, PA, USA; Institute for Diabetes, Obesity, and Metabolism, Perelman School of Medicine, University of Pennsylvania, Philadelphia, PA, USA; Penn/CHOP Kidney Innovation Center, Perelman School of Medicine, University of Pennsylvania, Philadelphia, PA, USA; Department of Genetics, Perelman School of Medicine, University of Pennsylvania, Philadelphia, PA, USA; Department of Dermatology, Perelman School of Medicine, University of Pennsylvania, Philadelphia, PA, USA; Renal, Electrolyte, and Hypertension Division, Department of Medicine, Perelman School of Medicine, University of Pennsylvania, Philadelphia, PA, USA; Institute for Diabetes, Obesity, and Metabolism, Perelman School of Medicine, University of Pennsylvania, Philadelphia, PA, USA; Penn/CHOP Kidney Innovation Center, Perelman School of Medicine, University of Pennsylvania, Philadelphia, PA, USA; Department of Genetics, Perelman School of Medicine, University of Pennsylvania, Philadelphia, PA, USA

**Keywords:** chronic kidney disease, fibrosis, GWAS, nephron progenitor differentiation, Notch signaling

## Abstract

**Background:**

Genome-wide association studies (GWAS) have identified numerous loci associated with kidney function, but linking non-coding variants to causal effector genes remains challenging. By integrating fine-mapped GWAS data from 2.27 million individuals with kidney expression and epigenomic datasets, we identified KDM1A as a top-prioritized gene at a kidney function locus. KDM1A is broadly expressed across nephron segments in mouse and human kidneys and is consistently upregulated in chronic kidney disease (CKD).

**Methods:**

We generated lineage-specific Kdm1a knockout mice targeting nephron progenitors (Six2-Cre) or differentiated tubular epithelial cells (Cdh16/Ksp1-Cre). Renal structure and function were assessed at baseline and following adenine-induced injury or unilateral ureteral obstruction using histology, immunoblotting, and gene expression analyses. Pharmacologic inhibition of KDM1A was evaluated in embryonic kidney explant cultures.

**Results:**

Nephron progenitor-specific deletion of Kdm1a resulted in severe developmental defects, including nephron loss, cystic degeneration, tubular atrophy, and early interstitial fibrosis. Mutant kidneys exhibited suppression of progenitor and segment-specific epithelial programs, loss of solute transporter expression, and derepression of developmental signaling pathways, including Notch and Wnt, accompanied by apoptosis and inflammation. In contrast, deletion of Kdm1a in differentiated tubular epithelial cells preserved baseline renal structure and function and did not modify fibrotic responses to injury. Pharmacologic inhibition of KDM1A during kidney development phenocopied genetic loss and led to accumulation of H3K4 methylation.

**Conclusions:**

KDM1A is a genetically supported, developmentally essential chromatin regulator required for nephron differentiation but dispensable in differentiated tubular epithelium. Its reactivation in CKD highlights a context-dependent epigenetic role and cautions against nonspecific KDM1A inhibition..

## Graphical Abstract

**Graphical Abstract:**
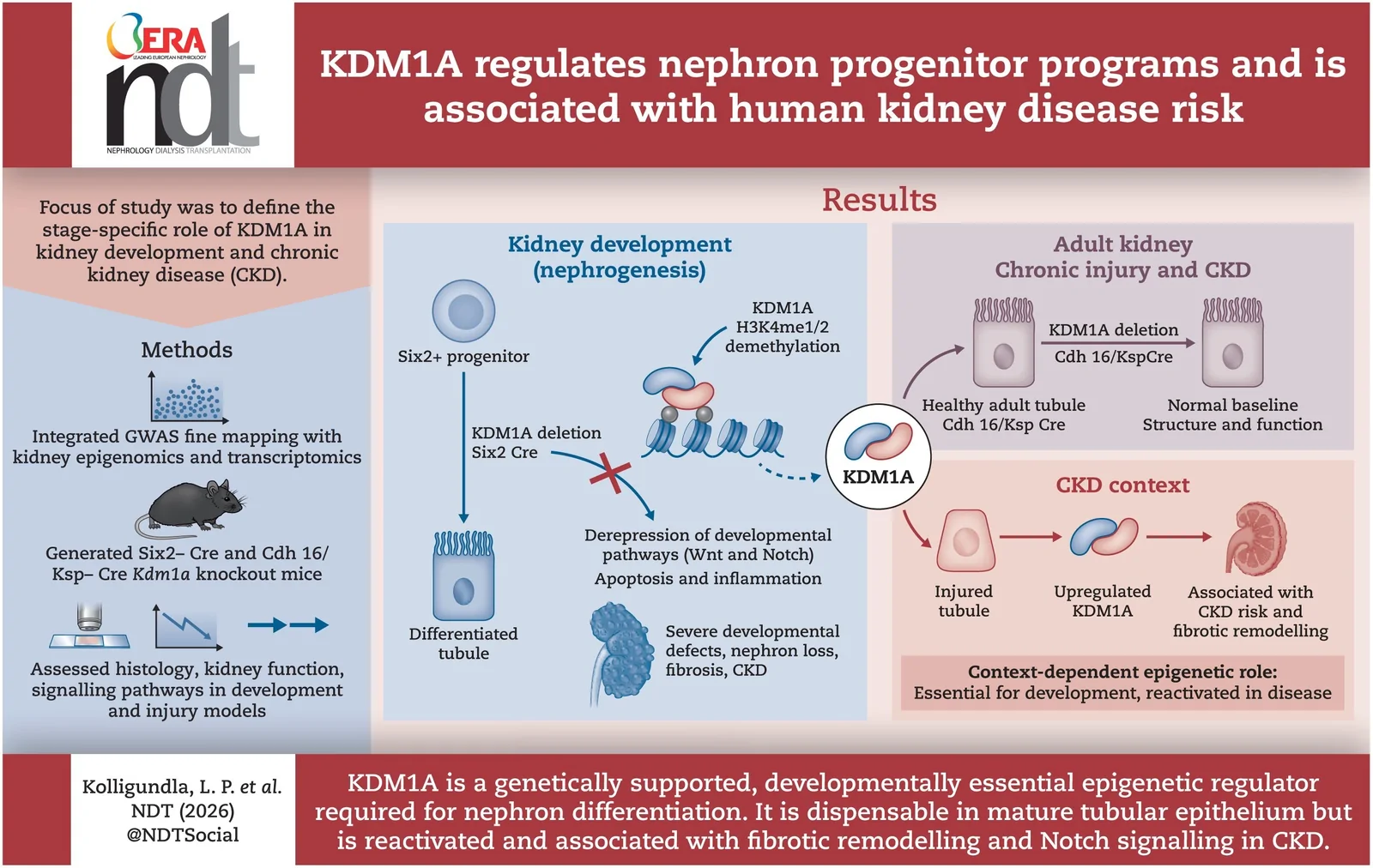

KEY LEARNING POINTS **What was known:**

- Genome-wide association studies (GWAS) have identified numerous loci linked to kidney function and chronic kidney disease (CKD), yet most reside in non-coding regions, leaving causal genes and mechanisms unresolved. Although epigenetic regulators contribute to kidney development and injury, the role of histone demethylases, including KDM1A, in kidney biology remained undefined.

**This study adds:**

- This study identifies KDM1A as a genetic effector gene at a kidney function GWAS locus on chromosome 1. KDM1A is required for nephron progenitor differentiation but is dispensable in differentiated epithelium after nephrogenesis. Six2-positive progenitor loss of Kdm1a causes impaired nephron formation, suppressed transport programs, derepressed Notch/Wnt signaling, and fibrosis.

**Potential impact:**

- These findings establish KDM1A as an epigenetic link between nephron development and CKD risk, providing a functional framework to interpret non-coding GWAS signals through chromatin regulation. Clinically, the results caution against non-specific KDM1A inhibition and emphasize the importance of stage-specific epigenetic modulation, illustrating how developmental regulators are repurposed during kidney injury.

## INTRODUCTION

Chronic kidney disease (CKD) affects nearly 10% of the global population and is a leading cause of morbidity and mortality worldwide. Despite advances in genetics, transcriptomics, and imaging, effective targeted therapies remain limited. A key obstacle is the incomplete understanding of molecular mechanisms governing kidney cell identity, differentiation, and maladaptive injury responses [1]. While studies of inflammation and fibrosis have identified important downstream pathways, the upstream regulatory layers coordinating developmental and disease programs remain poorly defined [2–7].

Epigenetic regulation including chromatin remodeling, histone modification, and DNA methylation has emerged as a central mechanism controlling gene expression in kidney development and disease. Epigenetic enzymes act as molecular switches that integrate environmental and genetic cues to maintain cellular identity and plasticity. Recent single-cell and spatial transcriptomic studies have revealed that epigenetic modifiers show striking cell-type and state-specific patterns across nephron segments, suggesting a critical yet underexplored role in renal physiology [8–10].

Genome-wide association studies (GWAS) have identified over a thousand genetic loci associated with kidney traits such as estimated glomerular filtration rate (eGFR), urine albumin-to-creatinine ratio, and electrolyte balance, encompassing data from more than 2 million individuals across diverse ancestries [11,12]. However, most signals map to non-coding regions, complicating causal gene and cell-type assignment. Integrative approaches combining epigenomic annotations with eQTL and meQTL data, the genetic “scorecard” strategy [13], have improved gene prioritization; yet most predicted regulatory loci, particularly those involving chromatin modifiers, remain functionally untested.

Among such regulators, lysine-specific demethylase 1A (KDM1A/LSD1) is a versatile epigenetic enzyme with context-dependent functions across tissues. KDM1A demethylates mono- and di-methyl groups from histone H3 lysine 4 (H3K4me1/2) and lysine 9 (H3K9me1/2), thereby fine-tuning enhancer and promoter activity [14]. Through its association with multi-protein complexes such as CoREST, NuRD, and NRSF/REST, KDM1A can function as either a transcriptional activator or a repressor [14,15]. KDM1A is essential for embryonic development, stem cell maintenance, and lineage specification, and its germline deletion causes early embryonic lethality [16].

In this study, we integrated genetic, transcriptomic, and epigenomic data to identify KDM1A as a gene of interest in kidney biology. We then used cell-type-specific knockout models to define its *in vivo* role. Deletion of Kdm1a in nephron progenitor cells (Six2-Cre) resulted in severe developmental defects, tubular atrophy, and fibrosis accompanied by aberrant activation of Notch and Wnt signaling pathways. In contrast, tubule-specific deletion (Cdh16/Ksp-Cre) in adult mice produced no overt phenotype under baseline or injury conditions, indicating a developmental stage-restricted requirement. Finally, human CKD samples revealed increased KDM1A expression and strong spatial co-localization with Notch pathway genes.

Together, these results identify KDM1A as an essential regulator of kidney development and a potential modulator of disease-associated signaling in chronic kidney injury.

## EXPERIMENTAL METHODS

### Gene prioritization strategy and genetic score card for KDM1A

Fine mapping analysis was performed to identify the potential causal variants within the KDM1A locus using CARMA [17]. Gene prioritization analysis for this locus was performed by integrating GWAS, epigenome data (snATAC-seq), and transcriptomic data to explore the regulatory variants and in association with multiple statistical tests, including Bayesian colocalization. Gene score card for the KDM1A involves 32 types of functional datasets, including coding variants, expression quantitative trait data (mQTL and eQTL) [18], allele-specific expression in tubule and glomerulus, fine-mapping credible sets, Bayesian colocalization, summary Mendelian Randomization (SMR), and the human kidney single-cell multi-omics ATAC-seq data, bulk-ATAC-seq, and ATAC-seq [13] based chromatin loop analysis, including Activity-by-Contact (ABC), Open4Gene, and Cis2Gene location. Human single-cell multi-omics ATAC-sequencing data was uploaded previously to CMDGA: https://cmdga.org/search/?type=Experiment&searchTerm=FNIH0000000.

### Generation of transgenic mice

#### Animal studies

The Institutional Animal Care and Use Committee at the University of Pennsylvania approved all animal-related protocols. The Kdm1A flox mice were obtained from Dr Brian Capell at Penn Epigenetic Institute, University of Pennsylvania. Kidney specific and tubule specific Kdm1A knockout mice were generated by crossing Kdm1A f/f mice with Ksp-Cre (stock #01223, Jackson) or Six2-Cre mice (stock #009606, Jackson) [19]. Parents and pups were genotyped for both Six2-Cre, wild-type, Ksp-Cre, and Kdm1A flox alleles by the KAPA Mouse Tail DNA Genotyping Kit. Mice were housed in a temperature- and humidity-controlled pathogen-free animal facility with a 12-h day and night cycle. Mice were provided with a standard chow diet (#5010, LabDiet) and water ad libitum.

#### Kidney explant culture—microdissection of embryonic kidneys and explant culture conditions

Embryonic kidneys were isolated using a previously described microdissection approach with minor modifications [20–22]. E15 embryonic stage was chosen to interrogate progenitor maintenance and epithelial patterning during kidney development [23]. E15 embryos were collected in ice-cold PBS; kidneys were dissected from the urogenital ridge under a stereomicroscope and transferred individually in cold PBS using wide-bore tips. Explants were cultured on membrane inserts in DMEM/F12 supplemented with 10% FBS and 1% penicillin/streptomycin, with inhibitors added as indicated. Kidneys were placed at the center of each insert, excess PBS was removed, and 500 μl medium was added to the lower chamber without contacting the membrane. Explants were cultured at 37 °C, 5% CO₂ for ∼72 h with daily medium changes.

### Statistics and reproducibility

Statistical significance was tested in the GraphPad Prism 10.5 software. One-way ANOVA and unpaired *t*-tests were performed to analyze the variables. No outliers were excluded from the analysis. The sample size per group in all experiments was determined based on the number of animals available in the colony of a specific age and gender. The number of replicates and sample size in each animal experiment were included in figures and/or figure legends. All data were expressed as means ± SEM or SD and stated in each figure legend. *P* values of <0.05 were considered statistically significant. The significance levels are indicated in the figures as follows: *P* < 0.05 (*), *P* < 0.01 (**), *P* < 0.001 (***), and *P* < 0.0001 (****).

## RESULTS

### Genome-wide association and multi-omics analyses implicate KDM1A as a kidney function gene

To identify candidate effector genes for kidney function, we examined a multi-ancestry GWAS of eGFR (eGFRcrea) comprising ∼2.27 million individuals [13]. At a locus on chromosome 1, multiple genome-wide significant variants spanned KDM1A, TCEA3, and ASAP3 (Fig. 1A). Fine-mapping identified two credible sets led by rs2806561 and rs754984, localizing to open chromatin near the KDM1A promoter and first intron, consistent with regulatory function (Fig. S1).

**Figure 1:**
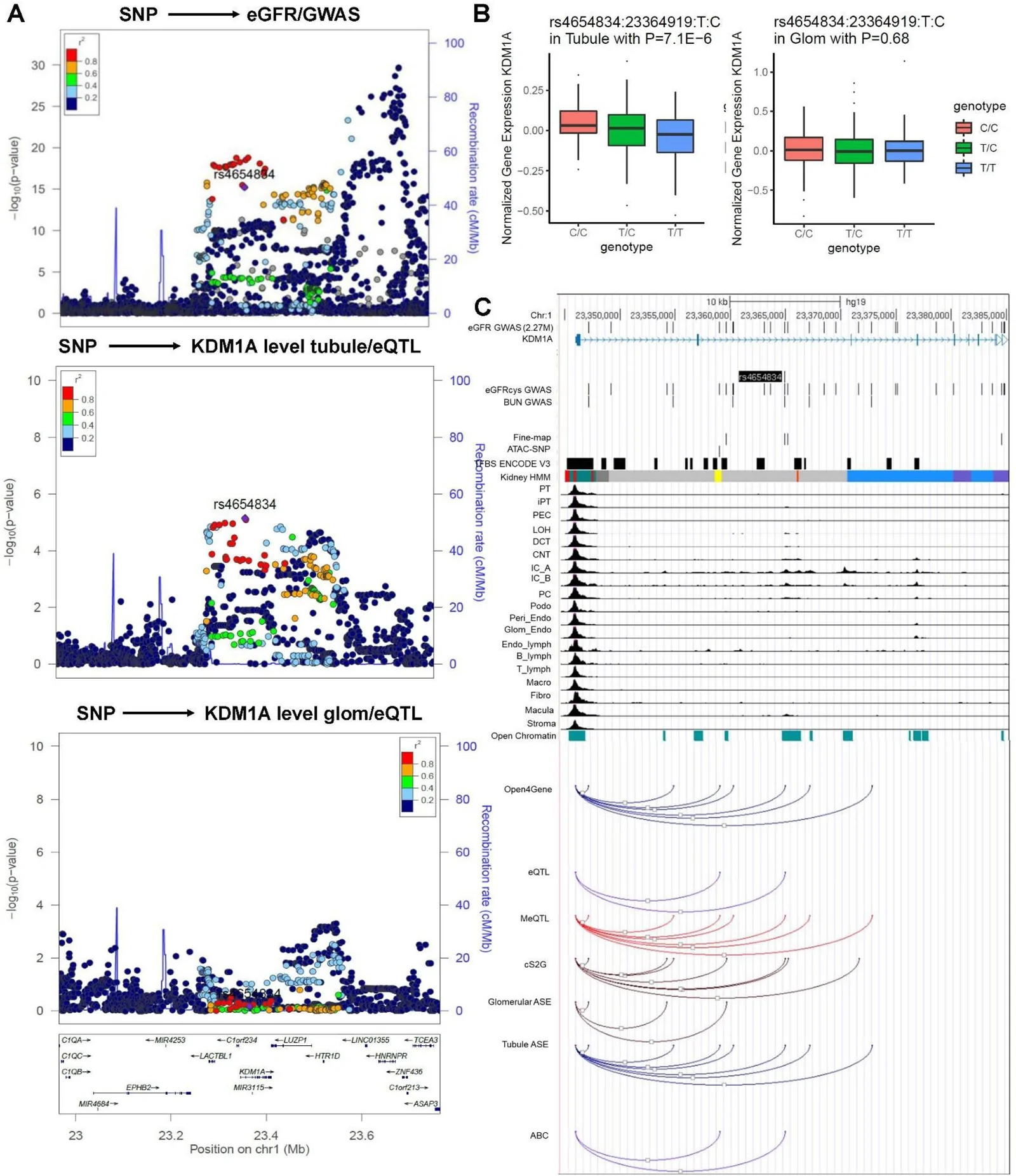
GWAS analysis implicates KDM1A as a kidney function gene. (A) Regional plot of *KDM1A* eGFR GWAS locus in European 1508 569 individuals (upper). The *x*-axis shows the chromosomal location with coding genes, and *y*-axis shows the strength of association [-log(*p*)] on chromosome 1. The variant (rs2806561) was selected as it is present in the *KDM1A* gene body and near its promoter. The color key indicates a correlation score (r2) with the top variant. Regional plot of genetic variants (rs4654834) associated with tubule (*n* = 356) (middle) glomerular (*n* = 315) (lower) specific quantitative traits. The *x*-axis indicates chromosomal location, and the *y*-axis shows the strength of the association [-log(*p*)] on chromosome 1. (B) KDM1A expression with respect to the variant rs4654384 in human kidney tubule (*n* = 356) and glomerulus (*n* = 315). (C) Multi-omic annotation of the regulatory variant (rs4654834). From top to bottom: GWAS LocusZoom, gene structure, annotation of variants, human kidney chromatin states, single-cell multi-omics ATAC, kidney open chromatin, transcription factor binding sites, kidney eQTL, tubule and glomerulus ASE, Cell2 gene (cS2G), and ABC.

To prioritize the causal gene, we integrated kidney compartment-specific eQTL and meQTL analyses with single-nucleus ATAC-seq. Sentinel variants strongly associated with KDM1A expression in both tubular and glomerular compartments, whereas neighboring genes demonstrated weaker or no colocalization (Fig. 1A and B). Variant-enriched regions overlapped accessible chromatin in nephron cell types relevant to eGFR, including proximal tubule and loop of Henle cells (Fig. 1C).

Chromatin interaction modeling, Bayesian colocalization, and summary-level Mendelian randomization further supported KDM1A as the effector gene, linking variant-containing enhancers to the KDM1A transcription start site. In a composite genetic scorecard, KDM1A ranked highest across all criteria, exceeding nearby candidates (Fig. 1C).

Together, these data identify KDM1A as the most likely effector gene at this kidney function locus, with non-coding variants regulating its expression via chromatin accessibility and long-range interactions.

### KDM1A is expressed throughout the nephron and increases in kidney injury

Double immunofluorescence staining was performed in healthy mouse and human kidney sections using segment-specific markers: *Lotus tetragonolobus* lectin (LTL) for proximal tubules, kidney-specific cadherin (KSP) for distal nephron segments, *Dolichos biflorus* agglutinin (DBA) for collecting ducts, and aquaporin 2 (AQP2) for principal cells. In both species, KDM1A was detected broadly across tubular epithelial segments, showing strong nuclear localization in epithelial cells consistent with its role as a chromatin-modifying enzyme (Fig. 2A and B). These findings indicate that KDM1A is a constitutive nuclear factor across the tubular epithelium rather than restricted to a specific segment.

**Figure 2:**
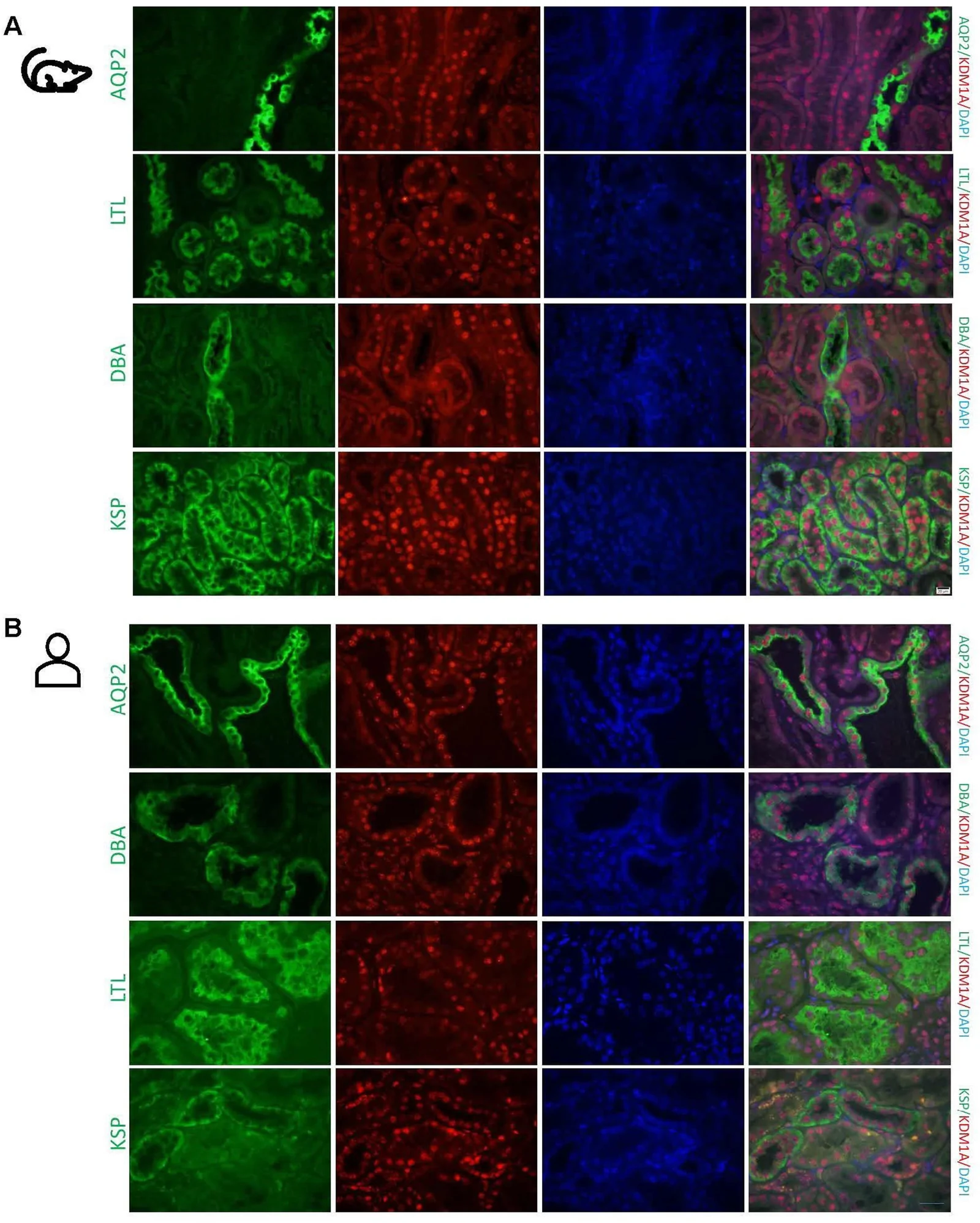
Kidney KDM1A protein expression and localization. (A) Immunofluorescence microscopy images of KDM1A with cell-type-specific markers such as LTL, KSP, DBA and AQP2. Scale bars, 20 μm (60×). (B). Immunofluorescence microscopy images of KDM1A with LTL, KSP, DBA, and AQP2 in healthy human kidney sections. Scale bars, 20 μm (60×).

Single-nucleus RNA-seq data from healthy mouse, rat, and human kidneys [9,10] confirmed widespread KDM1A expression across epithelial, endothelial, and stromal cells (Fig. S2A). Bulk RNA-seq analysis of multiple kidney injury models, including UUO, folic-acid nephropathy, APOL1-associated nephropathy, and TFAM knockout mice, revealed significant KDM1A upregulation in all models (log₂FC > 0.5, *P* < 0.01; Fig. S2B). Longitudinal gene expression analysis of KDM1A in adenine-induced CKD samples showed increased expression concomitantly with kidney injury and fibrosis markers (Fig. S3). Protein levels of KDM1A analyzed by immunofluorescence indicated increased KDM1A expression mainly in injured tubule cells without noticeable cytoplasmic-to-nuclear redistribution (Fig. S4), indicating dynamic regulation during injury.

### Nephron-progenitor-specific deletion of Kdm1a causes growth retardation, kidney dysfunction, cystic, and fibrotic remodeling

To investigate the developmental role of *Kdm1a*, we generated *Six2-Cre; Kdm1af/f* mice with nephron progenitor-specific deletion. *Six2-Cre* targets metanephric mesenchyme-derived nephron epithelia while sparing ureteric-bud-derived collecting system [24]. Immunofluorescence and immunoblotting confirmed near complete loss of KDM1A protein in *Six2* derived compartments, while expression persisted in collecting ducts (Fig. S5A and B).

*Six2-Cre; Kdm1af/f* mice exhibited marked postnatal growth retardation and small, pale kidneys (Fig. 3A). By 8 weeks, body and kidney weights were significantly reduced (*P* < 0.05) (Fig. 3B), accompanied by elevated BUN and serum creatinine, indicating impaired renal function (Fig. 3C). Injury markers, including *Lcn2* (*Ngal*) and *Havcr1* (*Kim1*) were induced, consistent with epithelial stress (Fig. 3D).

**Figure 3:**
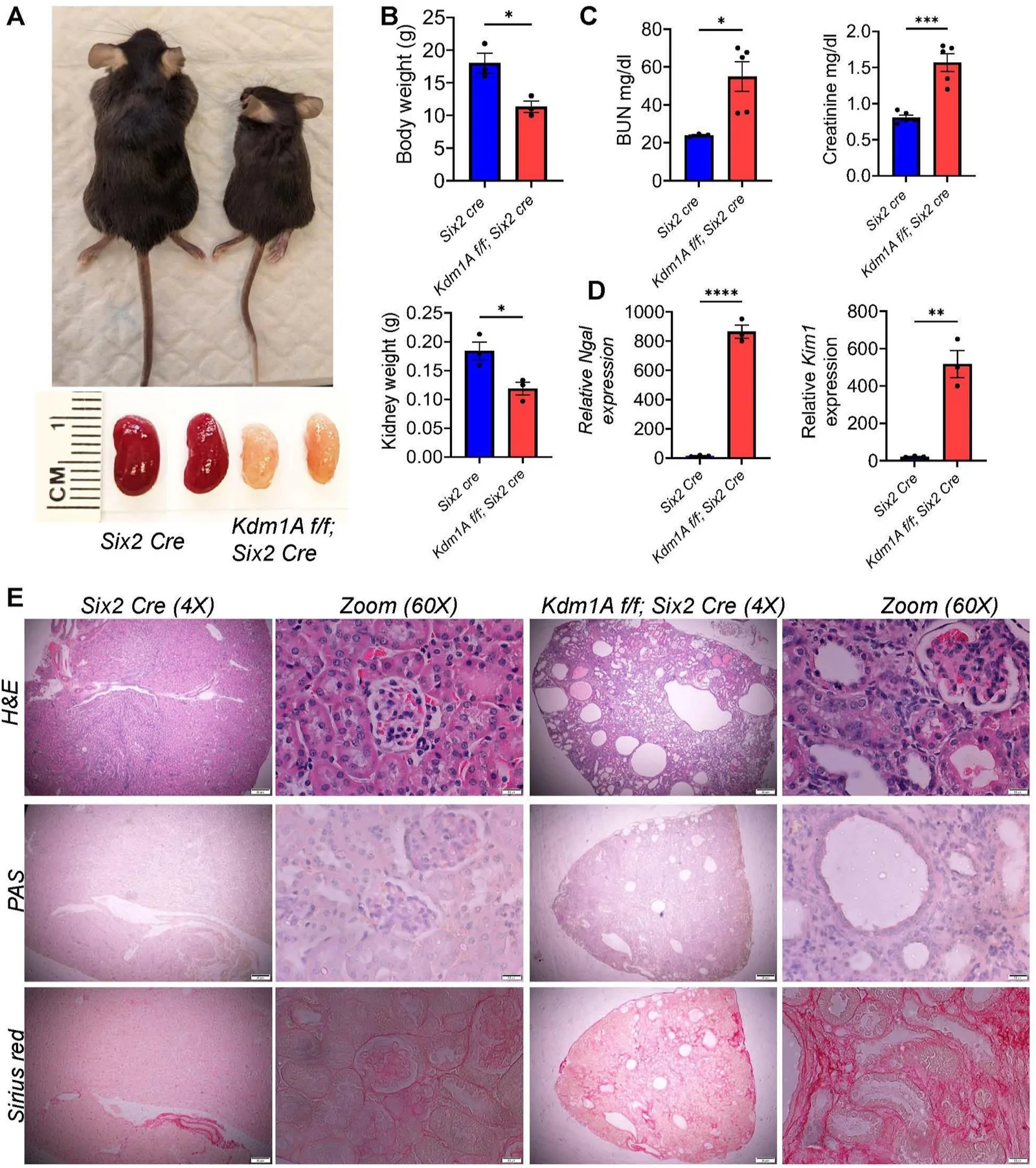
Morphological and histopathological changes in Kidney-specific deletion of KDM1A mice. (A) Comparative representation of *Six2-Cre Kdm1Af/f* vs *Six2-Cre* mice at 8 weeks of age (upper). Kidney pictures on a scale (lower). (Upper) Bar graphs report body weights (B), (lower) kidney weights, BUN and serum creatinine (C), gene expression of N-gal and Kim1 (D) in *Six2-Cre Kdm1Af/f; Six2-Cre* mice at 8 weeks of age *n* = 3-5 per group. (E) Microscopy images of Hematoxylin & eosin, periodic acid and Schiff’s (PAS), and Sirius red stainings in kidneys of 8 weeks *Six2-Cre Kdm1Af/f; Six2-Cre* mice. Scale bars, 200 μm (4×) and 20 μm (60×).

Histological analysis revealed profound architectural disruption, including tubular dilation, atrophy, cystic structures, and reduced glomerular number. Sirius Red staining demonstrated diffuse interstitial collagen deposition, confirming early fibrosis (Fig. 3E). Kidneys exhibited a characteristic cystic-fibrotic phenotype with immune infiltration and disrupted corticomedullary organization.

Together, these data demonstrate that KDM1A is essential for nephron development. Its loss in Six2-positive progenitors causes defective differentiation, nephron loss, fibrosis, and progressive renal failure, identifying KDM1A as a critical regulator of kidney structure and function.

#### Loss of KDM1A in nephron progenitors disrupts nephrogenesis and solute transport gene expression

To determine whether abnormalities in *Six2-Cre; Kdm1af/f* mice result from impaired nephrogenesis, we examined markers of nephron progenitor maintenance and differentiation. qPCR revealed reduced expression of glial cell-derived neurotrophic factor (*Gdnf*) and *Cited1*, two canonical markers of nephron progenitor cells, in *Kdm1a*-deficient kidneys, indicating defective progenitor maintenance or premature depletion (Fig. 4A).

**Figure 4:**
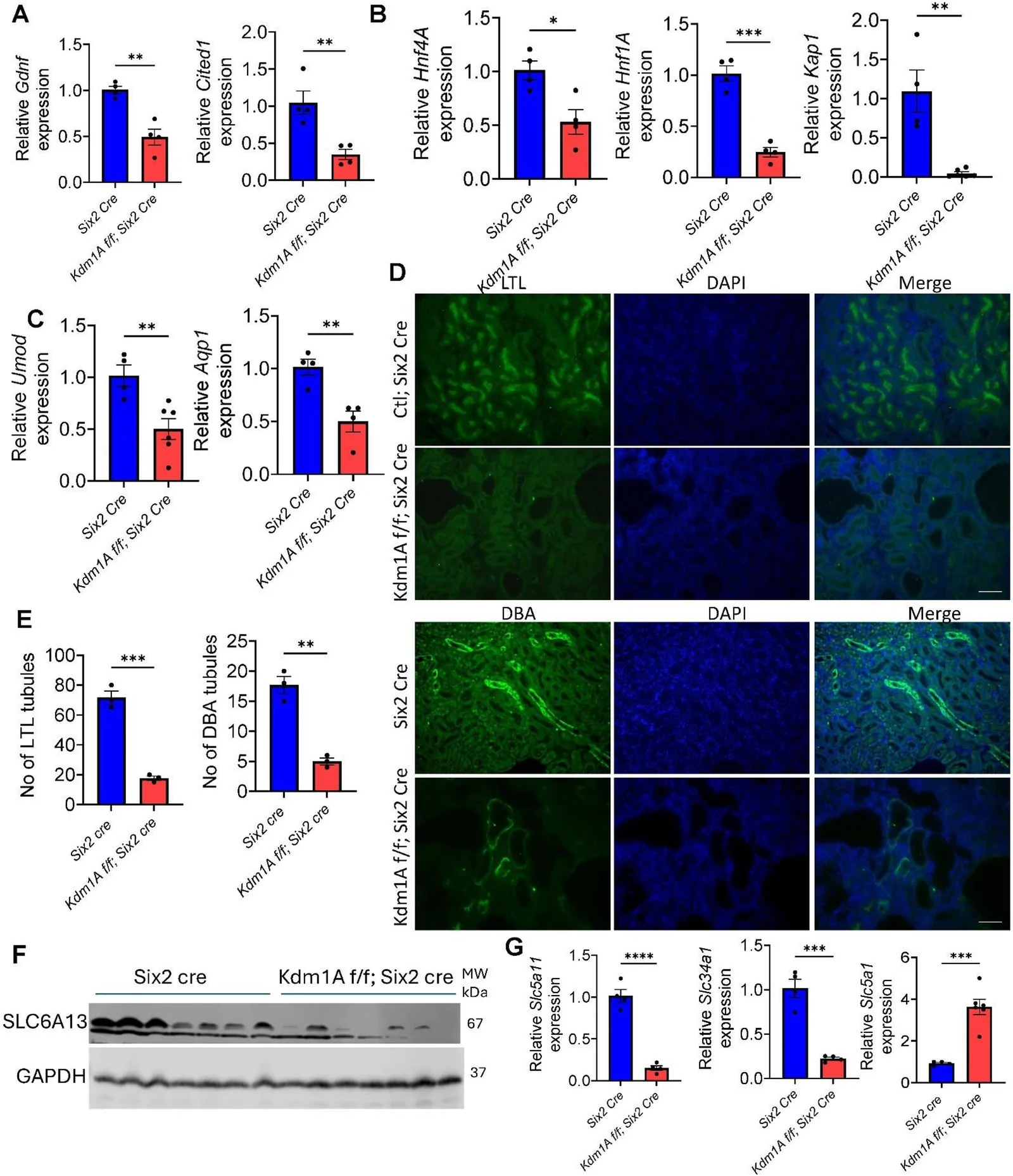
Gene expression analysis for nephrogenesis, tubule cell-specific and solute carriers in kidneys of Six2-Cre Kdm1Af/f; Six2-Cre mice. Bar graphs report transcript levels of glial derived neurotrophic factor (*Gdnf*), and Cited1 (A), hepatocyte nuclear factor 4A (*Hnf4A*), 1A (*Hnf1A*), and kidney androgen protein (KAP) (B), Uromodulin (*Umod*), and aquaporin 1 (Aqp1) (C) in kidneys of *Six2-Cre Kdm1Af/f; Six2-Cre* mice, *n* = 4. (D) Immunofluorescence microscopy images of LTL and DBA glycoproteins in kidney sections of *Six2-Cre Kdm1Af/f; Six2-Cre* mice. Scale bars, 50 μm (20×). (E) Bar graphs report quantification of LTL positive (left), and DBA positive (right) tubules in *Six2-Cre Kdm1Af/f; Six2-Cre* kidneys. (F) Immunoblots of SLC6A13 and GAPDH proteins in kidney lysates of *Six2-Cre Kdm1Af/f; Six2-Cre* mice. (G) Bar graphs report transcript levels of solute carriers *Slc5a11, Slc34a1*, and *Slc5a1* in *Six2-Cre Kdm1Af/f; Six2-Cre* mice, *n* = 4.

Markers of differentiated nephron segments were also decreased. Key proximal tubule transcription factors *Hnf4a* and *Hnf1a*, the S3-segment marker *Kap1*, as well as Uromodulin (*Umod*), a marker of the thick ascending limb, and Aquaporin 1 (*Aqp1*), were markedly downregulated in *Kdm1a*-deficient kidneys (Fig. 4B and C). These reductions indicate broad impairment of segment-specific epithelial differentiation across the nephron.

Immunofluorescence confirmed loss of tubular identity with fewer LTL (proximal tubule) and DBA (distal/collecting duct) positive structures, reduced brush border signal, and fewer tubular profiles in *Six2-Cre; Kdm1af/f* kidneys compared to controls (Fig. 4D and E).

Consistent with impaired epithelial maturation, solute transporter expression was reduced, including SLC6A13 protein and mRNA levels of *Slc34a1, Slc5a11*, and *Slc5a1* (Fig. 4F and G). Together, these findings demonstrate that KDM1A is essential for nephron differentiation and maturation, and its loss in nephron progenitors at developmental stages may disrupt segment specification, epithelial identity, and transport capacity, explaining the severe renal dysfunction observed in adult *Six2-Cre; Kdm1af/f* kidneys.

#### Loss of KDM1A in nephron progenitors derepresses developmental signaling pathways

Nephron progenitor-derived nephrogenesis is tightly regulated by canonical developmental cascades, including Wnt, Notch, and FGF signaling [25]. Given the severe structural abnormalities observed in *Six2-Cre; Kdm1af/f* kidneys, we investigated whether KDM1A loss affects these developmental programs. qPCR revealed increased *Notch1* and its targets *Heyl* and *Jag2*, and immunoblotting confirmed elevated NOTCH1 and NICD levels in *Kdm1a*-deficient kidneys (Fig. 5A and B).

**Figure 5:**
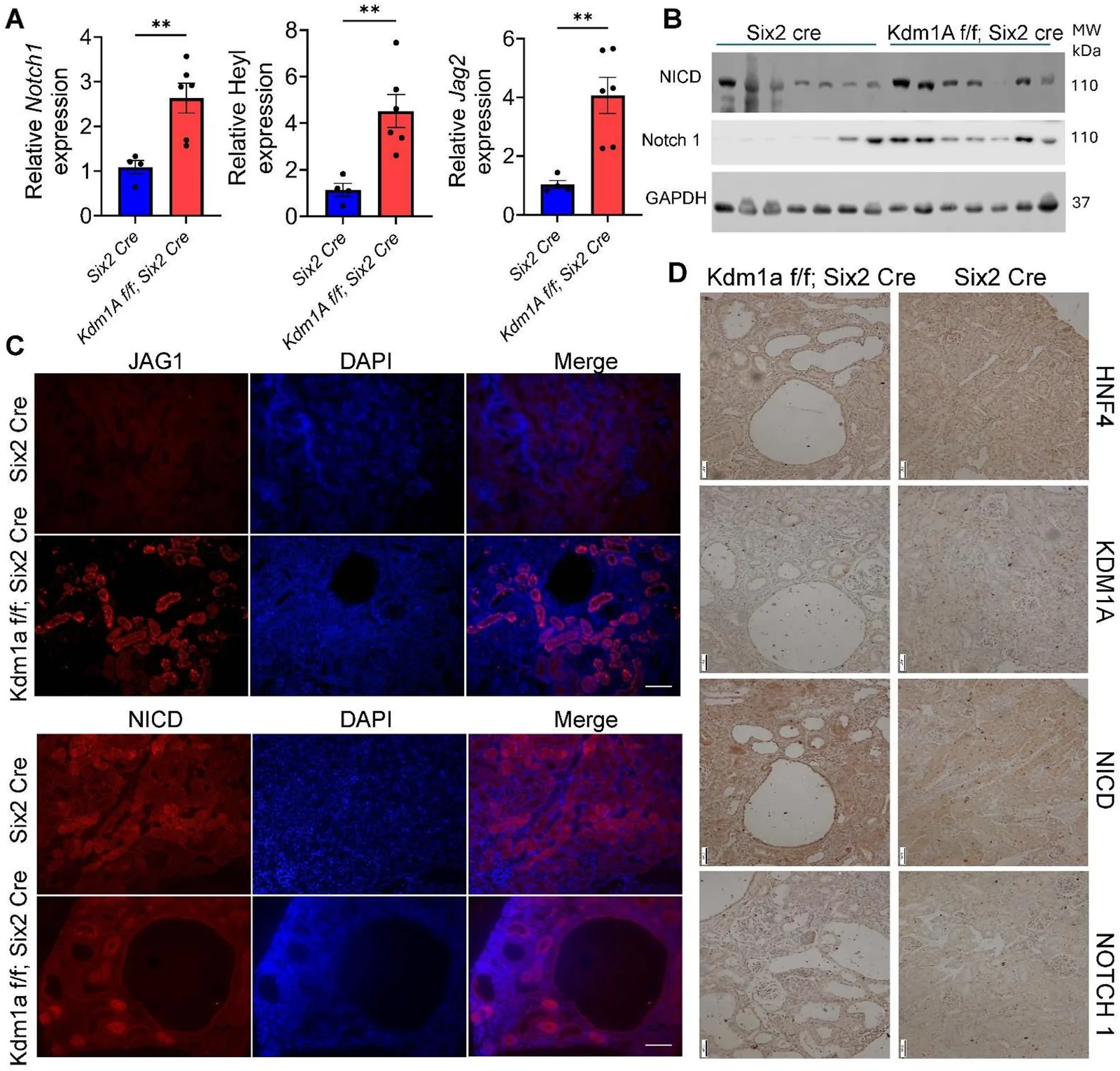
Gene expression and protein analysis of Notch pathway in kidney-specific deletion of KDM1A. (A) Bar graphs report transcript levels of *Notch1, Heyl*, and *Jag2* in kidneys of *Six2-Cre Kdm1Af/f; Six2-Cre* mice, *n* = 6. (B) Immunoblots of Notch intracellular domain (NICD), NOTCH1, and GAPDH proteins in kidney lysates of *Six2-Cre Kdm1Af/f; Six2-Cre* mice. (C) Immunofluorescence microscopy images of JAG1 and NICD in kidney of *Six2-Cre Kdm1Af/f; Six2-Cre* mice. Scale bars, 50 μm (20×). (D). Immunohistochemical micrsocopy images of HNF4A, KDM1A, NICD, and NOTCH1 in kidneys of *Six2-Cre Kdm1Af/f; Six2-Cre* mice. Scale bars, 50 μm (20×).

Immunofluorescence analysis further demonstrated intense NICD and JAG1 staining in the dilated tubular epithelium of *Six2-Cre; Kdm1af/f* kidneys, particularly in cystic regions (Fig. 5C). Immunohistochemical staining corroborated these findings, revealing elevated NOTCH1 and NICD proteins accompanied by a marked reduction in HNF4A, a transcription factor critical for proximal tubule differentiation (Fig. 5D). These findings indicate that KDM1A loss dysregulated Notch signaling, disrupting nephron differentiation and promoting cystic remodeling. Future single-cell and genome-wide chromatin profiling will be required to establish direct genomic binding or locus-specific regulation by KDM1A in nephron progenitors at molecular level.

#### Loss of KDM1A promotes apoptosis, inflammation, and fibrosis

Because Kdm1a-deficient kidneys showed severe tubular loss and cyst formation, we examined pathways related to cell death, inflammation, and fibrosis. Pro-apoptotic genes including *Bax, Bak, Bcl2*, and *Casp9* were markedly increased in *Six2-Cre; Kdm1af/f* kidneys, indicating enhanced apoptosis (Fig. 6A). Genes associated with pyroptosis and the cyclic GMP- AMP synthase-stimulator and interferon Gamma (cGAS–STING) pathway, including *Il10, Il18, Il1β, Isg20*, and *Isg15*, were also elevated compared to controls (Fig. 6B and C), indicating activation of inflammatory signaling.

**Figure 6:**
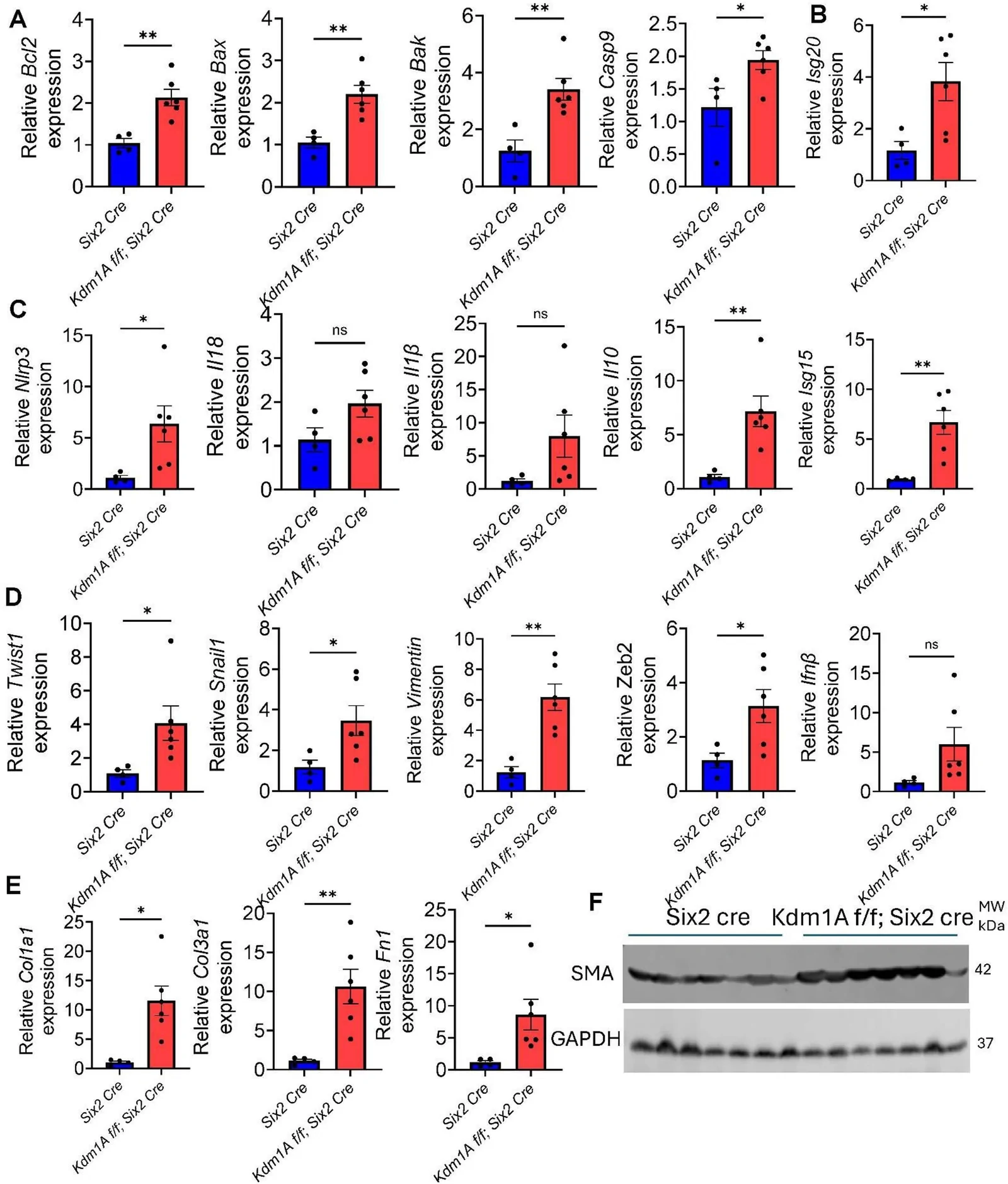
Gene expression analysis of apoptosis, inflammation, EMT, and fibrosis in kidney-specific deletion of KDM1A. Bar graphs report transcript levels of *Bcl2, Bax, Bak*, and *Casp9* (A), interferon-simulated genes (*Isg15* and *Isg20*), interferon β (*Ifnβ*) (B), pyroptosis genes inlcuding *Nlrp3*, interleukin 1β (*IL1β*), *Il18*, and *Il10* (C), EMT markers such as *Snail1, Twist1, Vimentin*, and *Zeb2* (D), fibrosis markers such as *Col1a1, Col3a1*, and *Fn1* in kidneys of *Six2-Cre Kdm1Af/f; Six2-Cre* mice, *n* = 6. (F) Immunoblots of αSMA and GAPDH in kidney lysates from *Six2-Cre Kdm1Af/f; Six2-Cre* mice.

Consistent with these changes, Kdm1a-deficient kidneys exhibited prominent fibrotic remodeling at baseline. Expression of *Col1a1, Col3a1*, and *Fn1*, as well as epithelial-to-mesenchymal-transition (EMT) markers *Snail1, Twist1, Zeb2*, and *Vimentin*, was increased (Fig. 6D and E), and immunoblotting confirmed elevated αSMA protein levels (Fig. 6F). Wnt signaling targets *Axin2, Lef1*, and *Wnt4* were also upregulated (Fig. S6), accompanied by histologic expansion of the interstitial matrix.

Together, these findings show that loss of KDM1A in nephron progenitors activates apoptotic, inflammatory, and developmental pathways, driving differentiation arrest, tubular loss, and fibrosis. This suggests that KDM1A acts as an epigenetic brake to maintain balanced signaling during nephron development and prevent maladaptive pathway reactivation.

#### Tubule-specific deletion of Kdm1a does not alter baseline or injury-induced kidney fibrosis

To assess KDM1A function in the adult kidney, we generated (Cdh16) Ksp-Cre; Kdm1af/f mice targeting distal nephron segments and collecting ducts. These mice were viable and showed normal kidney size, histology, and function at baseline, with no abnormalities on H&E or PAS staining (Fig. S7A and B). Immunostaining confirmed efficient loss of KDM1A protein in DBA-positive collecting duct cells, and not in LTL positive cells (Fig. S7C and D), without compensatory changes in KDM1B, KDM5A, or KDM5B expression (Fig. S8).

To assess whether KDM1A influences the response to injury, we subjected Ksp-Cre; Kdm1a f/f and wild-type littermates to unilateral ureteral obstruction (UUO) or adenine-induced chronic kidney injury. Following UUO or adenine injury, KDM1A expression was induced in wild-type kidneys, but distal nephron-specific deletion did not alter fibrosis. Expression of Col1a1, Col3a1, Fn1, αSMA, and fibronectin, as well as histologic fibrosis and tubular atrophy, was comparable between genotypes (Fig. 7A–D).

**Figure 7:**
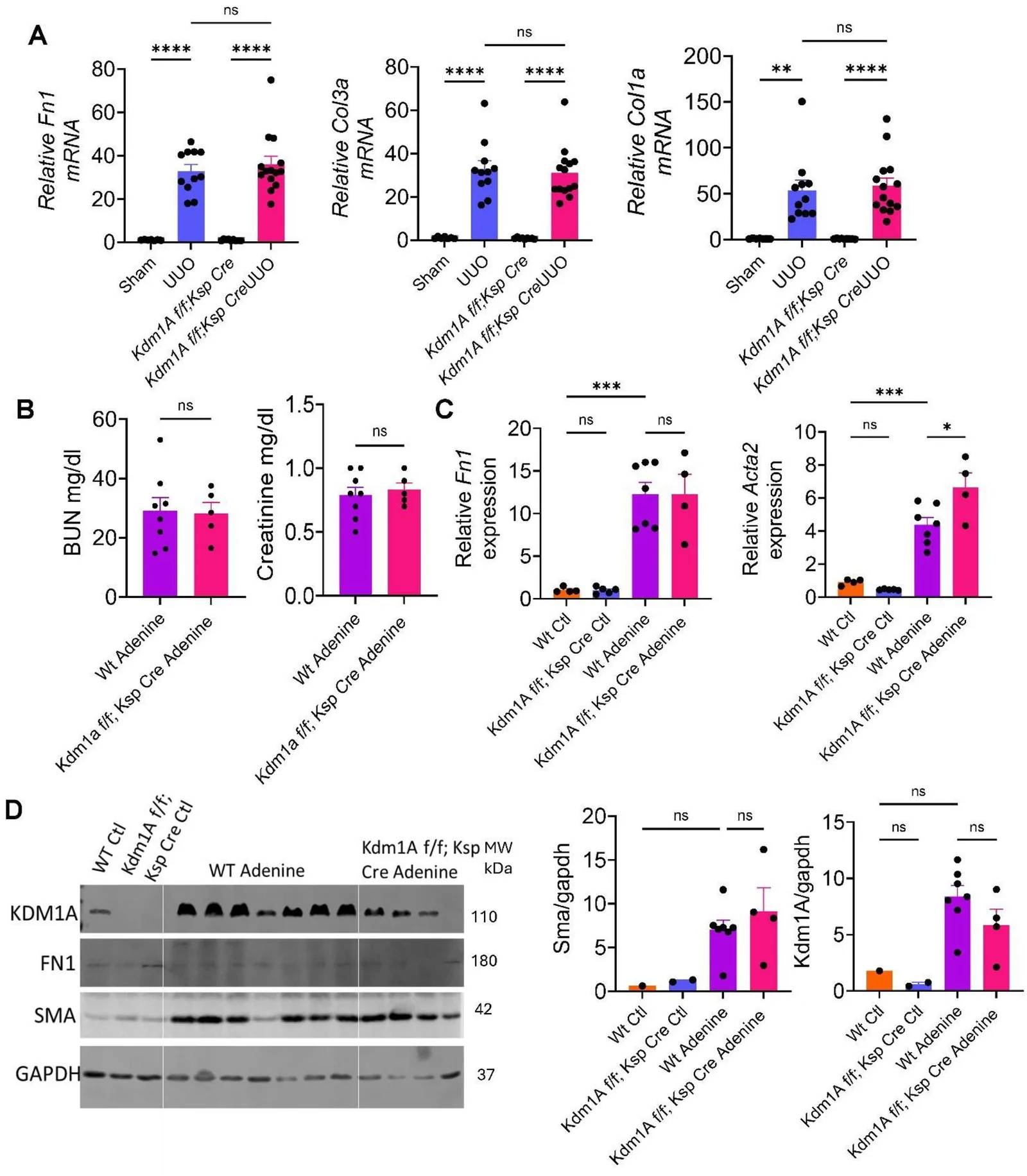
Tubule-specific *Kdm1A* deletion did not further elicit kidney injury response. (A) Bar graphs report transcript levels of *Col1a*, *Col3a*, and *Fn1 (A)* in kidneys of WT and *Ksp-Cre Kdm1Af/f* mice in Sham and UUO condition (Wt UUO *n* = 11 and *Ksp-Cre Kdm1Af/f* UUO *n* = 14). Bar graphs report blood urea nitrogen (left), serum creatinine (right) (B), transcript levels of Fn1, alpha smooth muscle actin (Acta2) (C) in WT and *Ksp-Cre; Kdm1Af/f* mice in adenine-fed condition, Wt adn *n* = 8 and *Ksp-Cre Kdm1Af/f* adn *n* = 4-5. (D) Immunoblots for KDM1A, fibronectin (Fn1), and SMA in kidneys of WT and *Ksp-Cre Kdm1Af/f* mice in adenine fed condition and their quantification.

Thus, although KDM1A is injury induced, its deletion in adult distal nephron segments does not affect baseline renal architecture or fibrotic responses, in contrast to the severe developmental defects observed in Six2-Cre; Kdm1af/f kidneys.

### KDM1A inhibition disrupts nephron epithelial organization during kidney development

To validate the developmental requirement for KDM1A and test whether enzymatic inhibition phenocopies genetic deletion, we treated E15 mouse kidney explants with the KDM1A inhibitor ORY-1001 for 72 h. Immunofluorescence was used to assess epithelial organization and progenitor distribution. Control explants formed well-organized nephron structures with intact epithelial tubes, sharply defined lumens, continuous epithelial cell adhesion molecule (EPCAM)-positive membranes, and preserved apical–basal polarity. SIX2-positive progenitor and early epithelial cells were uniformly distributed around developing nephron segments, consistent with normal nephron patterning (Fig. 8A).

**Figure 8:**
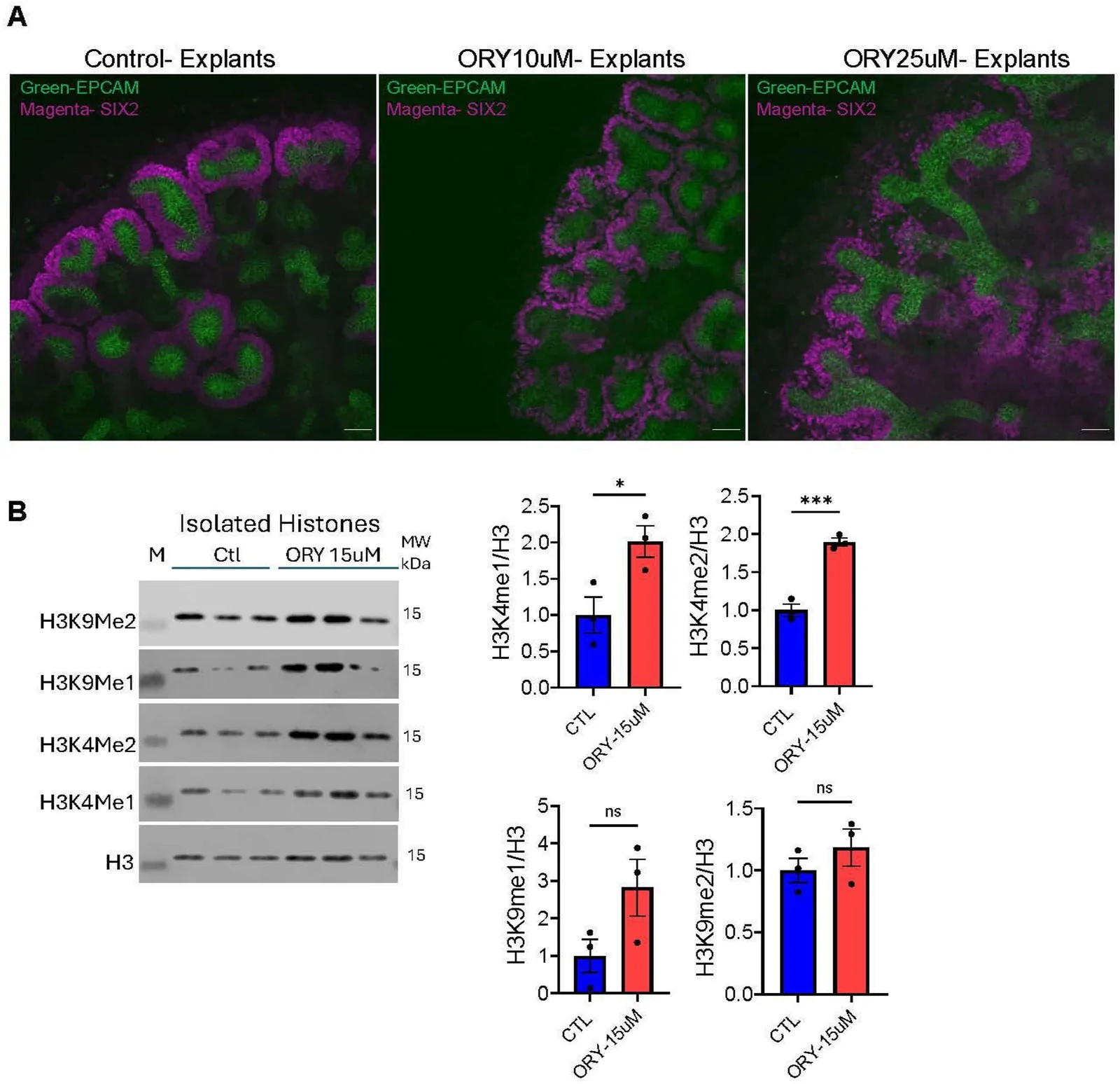
KDM1A inhibition disrupts nephron epithelial organization during kidney development and upregulation in global methylation levels in primary tubule cells. (A) Immunofluorescence microscopy images of E15 developing kidney explants cultured under three conditions: control medium (left), ORY 10 μM (middle), and ORY 25 μM (right). Explants are stained for EPCAM (green; tubular epithelial structures) and SIX2 (magenta; nephron progenitors). Scale bar, 50 μm (20×). (B) Western blots (left) and quantification bar plots (right) of histone methylation marks in primary mouse tubule cells treated with control or ORY 15 μM. Blots include H3K4me1, H3K4me2, H3K9me1, and H3K9me2, with total H3 shown as a loading control. An isolated histone fraction is shown for comparison.

In contrast, ORY-treated explants exhibited severe architectural disruption, with irregular, expanded, or collapsed tubules and disorganized EPCAM staining, indicating impaired epithelial maturation and polarity. SIX2-positive cells were abnormally clustered rather than evenly distributed, consistent with defective segmentation and progenitor organization. Thus, pharmacologic inhibition of KDM1A during development recapitulates the dysmorphogenesis observed in Six2-Cre; Kdm1af/f kidneys, demonstrating that KDM1A activity is required for proper epithelial patterning and nephron morphogenesis.

### Inhibition of KDM1A leads to global increase in histone methylation

To determine whether KDM1A inhibition alters global histone methylation consistent with its known enzymatic function, we performed immunoblotting for key histone modifications in primary mouse tubule cells treated with the KDM1A inhibitor ORY. KDM1A inhibition caused marked accumulation of H3K4me2 and a moderate increase in H3K4me1, confirming impaired H3K4 demethylation. In contrast, H3K9me1 and H3K9me2 levels were largely unchanged, indicating preferential targeting of H3K4 methylation in this context. Total histone H3 served as a comparable loading control across samples.

These results confirm the expected biochemical effects of KDM1A inhibition and demonstrate selective disruption of H3K4 methylation, a key chromatin modification regulating enhancers and promoters during kidney development (Fig. 8B).

## DISCUSSION

Our findings reveal that KDM1A/LSD1 serves a context-dependent and temporally restricted role in kidney biology, linking nephron progenitor differentiation to injury-associated chromatin responses. Through integrative genetic, transcriptomic, and open chromatin analyses, we identified KDM1A as a top-prioritized candidate at a kidney function-associated locus. Functional studies using conditional knockout models demonstrated that KDM1A is indispensable during kidney development, whereas its increased expression in adult CKD models appears to reflect a context-dependent response within injured epithelium rather than a primary initiating driver. Together, these observations define KDM1A as a developmental regulator whose dysregulation may contribute to kidney disease susceptibility.

Deletion of Kdm1a in Six2-positive nephron progenitors produced severe developmental defects characterized by growth retardation, tubular atrophy, cystic dilation, and early-onset fibrosis across various segments of the nephron. The phenotype reflects a failure of nephron progenitor cells to undergo coordinated differentiation, as evidenced by the marked downregulation of *Gdnf, Cited1, Hnf4a*, and *Umod* transcripts [26,27]. In parallel, solute transporter expression was suppressed, confirming loss of mature epithelial identity such as proximal and distal segments. These results position KDM1A as a critical regulator of nephron progenitor differentiation and epithelial maturation during development.

KDM1A’s canonical activity demethylation of H3K4me1/2 and H3K9me1/2 allows it to fine-tune the enhancer landscape of lineage-defining transcription factors. In other organ systems, such as brain, muscle, and hematopoietic tissues, KDM1A enforces lineage boundaries and represses aberrant activation of developmental programs. Our data are consistent with a similar role for KDM1A during kidney development; however, given the severity of the developmental phenotype, the transcriptional changes observed in mutant kidneys likely reflect a combination of primary epigenetic effects and secondary responses to impaired epithelial differentiation and tissue injury. In the absence of KDM1A, progenitors exhibit dysregulation of developmental signaling pathways, leading to disorganized differentiation and cystic malformation. Definitive identification of direct genomic targets of KDM1A in nephron progenitors will require cell-type-specific chromatin profiling approaches.

The upregulation of Notch1, NICD, and downstream targets (Hey1, Jag2, and Jag1) in Kdm1a-deficient kidneys indicates marked perturbation of developmental signaling balance [28]. Whether these alterations reflect direct chromatin-level regulation by KDM1A or secondary activation due to developmental arrest remains unresolved. This finding aligns with prior evidence that unrestrained Notch activation in nephron progenitors or mature tubules can induce cystic degeneration [29] and fibrosis [30]. Similarly, Wnt and EMT-related transcriptional changes observed in mutant kidneys suggest that KDM1A is required to stabilize differentiation programs during nephrogenesis.

These data fit into an emerging model in which epigenetic repressors such as KDM1A [31], DNMT1 [32], and KDM2A [33] function as “guardians of terminal differentiation.” When their activity is lost, developmental programs are aberrantly reactivated, leading to structural instability, epithelial injury, and fibrosis. Thus, the cystic and fibrosing phenotype of Six2-Cre; Kdm1af/f mice may reflect a convergence of developmental arrest and maladaptive signaling.

In contrast, deletion of Kdm1a in distal tubules and collecting ducts using Cdh16/Ksp-Cre produced no baseline phenotype and did not alter responses to UUO or adenine injury. These findings suggest that KDM1A is not required for maintenance of distal nephron structure under baseline conditions and does not substantially modify fibrotic remodeling in these injury models. This stage-specific requirement parallels observations in other tissues where KDM1A deletion in progenitors disrupts organogenesis, whereas deletion in differentiated cells results in a comparatively mild phenotype.

The lack of phenotype in adult deletion models may reflect the relative stability of chromatin landscapes once enhancer states are established during differentiation, reducing dependency on KDM1A in mature epithelial cells. Alternatively, functional redundancy among histone demethylases, including KDM1B or members of the KDM5 family, may buffer the loss of KDM1A in differentiated tubules [34,35]. Importantly, the absence of a strong phenotype in Cdh16/Ksp-Cre mice likely reflects lineage-restricted deletion within ureteric bud-derived distal segments, rather than a definitive lack of KDM1A function across all mature nephron compartments. Because proximal tubules are central mediators of injury in adenine nephropathy and UUO, a potential proximal tubule specific role of KDM1A cannot be excluded. In contrast, the pronounced Six2-Cre phenotype underscores the critical requirement for KDM1A during metanephric mesenchyme-derived nephron progenitor differentiation.

Future studies employing proximal tubule-specific deletion and integrative chromatin profiling will be required to define segment-specific and context-dependent roles of KDM1A in adult kidney disease. Another limitation is that the current study mainly focused on histopathological changes rather than metabolic and electrolyte balance despite severe defects in nephrogenesis and solute carriers’ expression. This will be addressed in our future segment-specific KDM1A knockout mice.

Species-specific differences in KDM1A expression and developmental timing may also influence functional dependency. Public datasets indicate relatively low bulk KDM1A transcript abundance in human fetal kidney, whereas adult human kidneys demonstrate broad tubular and glomerular expression, including in podocytes and endothelial cells [36]. These patterns suggest potential differences in compartmental reliance on KDM1A that are not fully captured by lineage-restricted mouse models will require complementary approaches such as human organoid systems, spatial transcriptomics, or genetic perturbation models.

Although developmental deletion of KDM1A results in nephron deficiency, inhibition of KDM1A with ORY1001 repressed nephron development in mouse kidney explants. Time-course analyses in adenine-induced CKD indicate that Kdm1a upregulation occurs concomitantly with established injury markers rather than preceding them, supporting interpretation as an injury-associated response. This apparent paradox mirrors a common theme among epigenetic regulators: genes indispensable for organogenesis are often reactivated in chronic injury [32,37,38].

These findings may have translational implications. KDM1A inhibitors are currently under clinical evaluation in oncology and neurodegenerative disorders [39]. However, the present data caution that systemic inhibition of KDM1A may disrupt renal development or exacerbate epithelial vulnerability, particularly in pediatric settings or regenerative contexts. A deeper understanding of KDM1A’s chromatin targets, cofactor interactions, and stage-specific functions will be essential to safely harness this pathway therapeutically.

In summary, this study establishes KDM1A as a pivotal regulator linking nephron development to CKD risk. During embryogenesis, KDM1A is required for coordinated nephron differentiation and is associated with stabilization of developmental signaling programs; in adulthood, its induction accompanies injury-associated chromatin remodeling without demonstrating a primary disease-driving role in distal tubules. These dual roles underscore the importance of distinguishing developmental versus adaptive functions of chromatin regulators when considering therapeutic strategies.

## Supplementary Material

gfag073_Supplemental_File

## Data Availability

The GWAS and QTL data are publicly available at https://susztaklab.com/. All other data needed to evaluate the conclusions in the paper are present in the paper and/or the Supplementary materials.
