# Supplementary material for: KDM1A regulates nephron progenitor programs and is associated with human kidney disease risk
Source: Nephrol Dial Transplant. 2026 May 1;41(11):2027–41. doi: 10.1093/ndt/gfag073 (PMC13719706; doi:10.1093/ndt/gfag073)
Supplement: Supplementary file 1 [file gfag073_supplemental_file.docx]

**KDM1A regulates nephron progenitor programs and is associated with human kidney disease risk**

Lakshmi P Kolligundla^1,2,3,4,#^, Dhanunjay Mukhi^1,2,3,4,#^, Jonathan Levinsohn^1,2,3,4^, Andi Bergerson^1,2,3,4^, Yanjuan Hou^1,2,3,4^ , Hongbo Liu^1,2,3,4^, Katalin Susztak^1,2,3,4^*

\# These authors equally contributed to this work

**This file includes**

**Supplementary methods**

**Supplementary Figures**

**fig. S1.** Fine mapping in the 1.7M European population and the newest prioritisation from the Genetic Scorecard concept.

**fig. S2.** KDM1A expression in multi-species snRNA seq and RNA seq in mouse kidney disease models.

**fig. S3.** Transcript levels of KDM1A and kidney injury and fibrosis markers.

**fig. S4.** KDM1A expression in Adenine induced CKD model**.**

**fig. S5.** KDM1A expression in *Six2 Cre Kdm1A f/f* mice.

**fig. S6.** WNT pathway target genes in Kdm1A knockout kidneys.

**fig. S7.** Mice with tubule-specific KDM1A loss have no baseline kidney function defects.

**fig. S8.** Transcript levels of KDM family members.

**Supplementary table**

**Table 1. Mouse primers table**

**Supplementary methods**

**Animal experiments:**

8 weeks old Six2 Cre Kdm1a f/f mice were used in this study to observe adult kidney phenotype. Kidney injury models such as UUO and adenine-induced CKD were only applied in tubule-specific Kdm1A knockout mice. Male mice were used for adenine-fed CKD, whereas both male and female mice were used for UUO-induced kidney fibrosis. Seven-week-old male C57BL/6 mice were randomly assigned to control and experimental groups. UUO kidney fibrosis was induced by ligating the left kidney ureter with a nylon string, while the right kidney served as a sham control. UUO mice were sacrificed on 7-days of post-surgery. Kidney tissues were collected for histology and molecular studies.

7-8 weeks-old littermate male mice were selected to feed with 0.2% adenine diet (#TD.160020, Inotiv, USA) for 28 days, and age-matched male littermates served as controls, as they were fed with a casein calcium phosphate diet (#TD.150303, Inotiv, USA). Animals were sacrificed, and blood samples and tissues were harvested for histology, molecular and biochemical analyses.

**Kidney function measurements**

Serum was collected through retro-orbital blood vessels. Serum creatinine (#DZ072B-KY1, Diazyme), blood urea nitrogen (#B7552150, Pointe Scientific) according to the manufacturer's protocol.

**Gene expression analysis**

About 20 mg of kidney tissue was homogenised in 1ml of Trizol (Ambion) followed by 200μl of chloroform addition to this homogenate, briefly vortexed and incubated for 3 minutes at room temperature. Then, samples were centrifuged at a top speed and collected the top layer was collected in a new tube. An equal volume of 100% isopropanol was added to the top layer, and the tubes were centrifuged again at a top speed at 4°C. Then the RNA pellet was washed with 70% ethanol, dried, and resuspended in clean RNase-free water and treated with DNase I before proceeding with cDNA conversion.

A total of 2μg RNA was converted into cDNA with the High-Capacity cDNA Reverse Transcription Kit (#4368813, Applied Biosystems). Real-time PCR analysis was performed in the Applied Biosystems Quant Studio 5 system and quantified the relative fold change by the 2^-ΔΔCT method. The primer sequences are listed in Supplementary Table 1.

**Western blotting**

About 15-20mg of kidney tissue was homogenised (#LR60902, OMNI-GLH) in SDS-blue loading buffer (#7722, CST) with 42 mM DTT. The protein lysate was sonicated (Diagenode), heated at 95°C, and cooled down for 5min on ice. After brief centrifugation at high speed for two minutes, the supernatants were loaded and separated on SDS-PAGE gels, then transferred onto a PVDF membrane at 100 V on ice. The PVDF membranes were blocked in 5% non-fat dry milk powder in tris-buffer saline containing 0.1% Tween-20 (TBST) solution for 30 minutes at room temperature, and incubated with primary antibodies overnight at 4°C. After the primary antibody incubation, the blots were washed in TBST and probed with IRdye-conjugated or HRP-conjugated secondary antibodies for an hour at room temperature. Finally, the blots were scanned at excitation wavelengths of 600, 700, and 800 or chemiluminescence using a Li-COR imager (Odyssey® Fc) and analysed using Image Studio software. Blot quantifications were performed in Image J software.

The Primary antibodies were used for western blots in this study: slc6a13, PA5-95662; NICD, ab52302; Notch1, 3608S; GAPDH, MA1-16757; KDM1A, ab129195; JAG1, sc6011; SMA, MA1-06110; FN1, PA5-99457; H3K9me1, ab176880; H3K9me2, ab176882; H3K4me1- ab176877; H3K4me1, ab7766.

**H&E, Sirius Red and Periodic acid Schiff’s (PAS) staining**

To do these three staining procedures, first, paraffin-embedded formalin-fixed kidney sections were heated at 65°C for an hour and then proceeded with rehydration steps from xylenes to alcohol gradient steps (100% to 70%). Then, slides were proceeded with H&E or Sirius red or PAS staining as previously discussed. Images were captured in an Olympus 5000 microscope equipped with Cell Sense software at 4X, and 60X magnification (scale bar=200µm and 20µm).

**Immunofluorescence and Immunohistochemical staining**

Five-micrometer FFPE kidney sections were deparaffinized, rehydrated, subjected to citrate-based antigen retrieval, and blocked in PBS containing 10% BSA and 0.1% Tween-20. Sections were incubated overnight at 4 °C with primary antibodies: anti-KDM1A (1:50, Abcam #AB129195), LTL-Fluorescein (1:200, Vector Labs #FL-1321), anti-KSP (1:50, Santa Cruz #sc-393153), DBA (1:50, Santa Cruz #sc-9882), anti-NOTCH1 (Cell Signaling #3608S), NICD (Abcam #AB52302), Kim1( R&D systems #AF1817); SIX2 (Proteintech #11562-1-AP) and JAG1 (Santa Cruz #sc6011). Sections were then incubated with Alexa Fluor 555–conjugated secondary antibody (1:400, Invitrogen #A21432) and mounted with DAPI antifade medium (Invitrogen #P36941).

For immunohistochemistry, sections were processed similarly through blocking, treated with avidin–biotin, incubated with HRP-conjugated secondary antibodies, and developed using ImmPACT DAB. Images were acquired on an Olympus BX5000 microscope at 20× or 60× magnification.

**Supplementary Figures**

**Supplementary Figure 1:** Fine mapping in the 1.7M European population and the newest prioritization from the Genetic Scorecard concept.

A. Fine mapping analysis of the eGFR GWAS locus in 1.7 million European individuals. The X-axis indicates the chromosomal location. The upper panel shows GWAS associations with eGFRcrea, and the y-axis shows the strength of the association (-log(p)). The second panel shows the credible sets fine-mapped by CARMA, and the Y-axis shows the posterior inclusion probability (PIP). Bottom panels show the GWAS associations with different index variants from two credible sets. Each dot is one SNP. Red color representing squared correlation (r2) between index variant and other variants and the gray color dots are non-correlated SNPs.
B. Genetic Scorecard for KDM1A. Score Card concept consists variant score, gene score and target score for the same GWAS locus. The GWAS dataset is from a 2.27M multi-ethnic population.

**Supplementary Figure 2:** KDM1A expression in multi-species snRNA seq and RNA seq in mouse kidney disease models.

A. *Kdm1A* expression in mouse (left), rat (middle), and human (right) kidney single-nucleus RNA sequencing.
B. *Kdm1A* expression in bulk kidney RNA sequencing in UUO, folic acid, TFAM knockout and APOL1 transgenic mice.

**Supplementary Figure 3:** Transcript levels of KDM1A and kidney injury and fibrosis markers. *Kdm1A, Kim1, Ngal, Fn1* and *Acta2* expression levels in time dependent manner of adenine induced CKD model.

**Supplementary Figure 4:** KDM1A expression in Adenine induced CKD model**.**

A. Double-Immunofluorescence for DBA and KDM1A in kidney sections of WT control and WT Adenine. Scale bars, 50 μm (20X).
B. Double-Immunofluorescence for LTL and KDM1A in kidney sections of WT control and WT Adenine. Scale bars, 50 μm (20X).
C. Double-Immunofluorescence for KIM1 and KDM1A in kidney sections of WT control and WT Adenine. Scale bars, 50 μm (20X).

**Supplementary Figure 5:** KDM1A expression in *Six2 Cre Kdm1A f/f* mice.

A. Immunofluorescence of KDM1A in kidney of *Six2 Cre Kdm1Af/f*; *Six2 Cre* mice. Scale bars, 50 μm (20X).
B. Immunoblots of KDM1A and GAPDH in kidney lysates of *Six2 Cre Kdm1Af/f*; *Six2 Cre* mice.

**Supplementary Figure 6:** WNT pathway target genes in Kdm1A knockout kidneys. Transcript levels of *Axin2, Lef1* and *Wnt4* were measured in the kidneys of *Six2 Cre Kdm1Af/f*; *Six2 Cre* mice.

**Supplementary Figure 7:** Mice with tubule-specific KDM1A loss have no baseline kidney function defects.

D. Baseline serum creatinine (sCr), and blood urea nitrogen (BUN) measured in WT and *Ksp Cre Kdm1Af/f* mice.
E. Haematoxylin &Eosin (H&E), Periodic acid and Schiff’s (PAS), and Sirius red staining in kidneys of WT and *Ksp Cre Kdm1Af/f* mice. Scale bars, 50 μm (20X).
F. Double-Immunofluorescence for LTL and KDM1A in kidney sections of WT and *Ksp Cre Kdm1Af/f* mice at baseline (right). Quantification of tubule number positively stained for LTL, and KDM1A (left). Scale bars, 50 μm (20X).
G. Double-Immunofluorescence for DBA and KDM1A in kidney sections of WT and *Ksp Cre Kdm1Af/f* mice at baseline (right). Quantification of tubule number positively stained for DBA and KDM1A (left). Scale bars, 50 μm (20X).

**Supplementary Figure 8:** Transcript levels of KDM family members. *Kdm1A, Kdm1B, Kdm5A*, and *Kdm5B* in WT and *Ksp Cre Kdm1A f/f* mice kidneys.

**Supplementary Table 1**

Mouse primers were used in this study.

| S.No | Gene | Forward primer | Reverse primer |
| --- | --- | --- | --- |
| 1 | *Kdm1A* | AGCTACAGAGTTTTGGGATGG | GATTCCCTCCAAGACCTGTTAC |
| 2 | *Kdm1B* | AAACCTCATCCTCGCACTG | GAATCCTCTCCACTTCCTGAAC |
| 3 | *Kdm5A* | CTCCTGTAATCCGGAGCGAC | GGCTGATGACACACGGAAGA |
| 4 | *Kdm5B* | AGATGGGTTTTGCTCCTGG | TCCTTGTCCTTTGTGTCGG |
| 5 | *Gdnf* | TGAAGACCACTCCCTCGG | GCTTGTTTATCTGGTGACCTTTTC |
| 6 | *Cited1* | AACCTTGGAGTGAAGGATCGC | GTAGGAGAGCCTATTGGAGATGT |
| 7 | *Hnf4a* | CCCTGGAGTTTGAAAATGTGC | AGGCTGTTGGATGAATTGAGG |
| 8 | *Hnf1a* | ACACCCATGAAGACACAGAAG | TCTTAGTTGGCAGCTCATCG |
| 9 | *Kap1* | AATACACCAAGGACCACACTG | CTCTCACAGAACAGCACGAG |
| 10 | *Umod* | CTTACTGCACCGATCCTAGTTC | CTCCAGCCTGTACTCCAATTG |
| 11 | *Aqp1* | CTGGCGATTGACTACACTGG | AAGTCATAGATGAGCACTGCC |
| 12 | *Col1a1* | TGCCTGGACCTCCTGGCGAGCGT | AGCAGGTCCGGGAGCACCACGTT |
| 13 | *Col3a* | ACAGCTGGTGAACCTGGAAG | ACCAGGAGATCCATCTCGAC |
| 14 | *Fn1* | ACAAGGTTCGGGAAGAGGTT | CCGTGTAAGGGTCAAAGCAT |
| 15 | *Acta2* | GTTCAGTGGTGCCTCTGTCA | ACTGGGACGACATGGAAAAG |
| 16 | *Slc5a11* | ACATCGCTGTCCTAGTTCTGT | TGGCGAACAAGGATGGACC |
| 17 | *Slc34a1* | CTCATTGTGGGTGCCCAACATGAT | ACCATGTGTCTCCCACGGACTGGAAG |
| 18 | *Slc5a1* | CTTTGAATGGAACGCCTTGG | GGACAGAACGGAAAGGTAGATC |
| 19 | *Notch1* | ACAGTGCAACCCCCTGTATG | TCTAGGCCATCCCACTCACA |
| 20 | *Heyl* | TTCGGCATGAAGCGGCCCAG | CTCCAGCTTGGAGGAGCCCTGT |
| 21 | *Jag2* | CGACTCACACTGCGCTTCA | TCGGATTCCAGAGCAGATAGC |
| 22 | *Havcr1* | TCCACACATGTACCAACATCAA | GTCACAGTGCCATTCCAGTC |
| 23 | *Lcn2* | GCCCTGAGTGTCATGTGTCT | GAACTGATCGCTCCGGAAGT |
| 24 | *Vimentin* | GGAGGCCACGAACTTCACTCT | GGGATGCAACACCTATTGTCAGT |
| 25 | *Bcl2* | GGACTTGAAGTGCCATTGGTA | GTTATCATACCCTGTTCTCCCG |
| 26 | *Bax* | CTGCAGAGGATGATTGCTGA | GAGGAAGTCCAGTGTCCAGC |
| 27 | *Bak* | TGATATTAACCGGCGCTACG | AGCTGATGCCACTCTTAAATAGG |
| 28 | *Casp9* | GTCAAGTTTGCCTACCCCCA | GAGCCCACTGCTCCAGAATG |
| 29 | *Isg20* | GTATGACAAGTACATCCGACCC | ACCAGCTTGCCTTTCAGAAG |
| 30 | *Nlrp3* | ATTACCCGCCCGAGAAAGG | TCGCAGCAAAGATCCACACAG |
| 31 | *Il18* | ACTGTACAACCGCAGTAATAC | AGTGAACATTACAGATTTATCCC |
| 32 | *Il1β* | GCAACTGTTCCTGAACTCAACT | ATCTTTTGGGGTCCGTCAACT |
| 33 | *Il10* | AGCCGGGAAGACAATAACTG | GGAGTCGGTTAGCAGTATGTTG |
| 34 | *Isg15* | CTAGAGCTAGAGCCTGCAG | AGTTAGTCACGGACACCAG |
| 35 | *Twist1* | CTCAGCTACGCCTTCTCC | ACTGTCCATTTTCTCCTTCTCTG |
| 36 | *Snail1* | TGGAAAGGCCTTCTCTAGGC | GGAGAATGGCTTCTCACCAG |
| 37 | *Zeb2* | GGAGCTAAGGGAGAGTGTTG | AATTGTGGTCTGGATCGTGG |
| 38 | *Ifnβ* | CGAGCAGAGATCTTCAGGAAC | TCACTACCAGTCCCAGAGTC |
| 39 | *Lef1* | TGTTTATCCCATCACGGGTGG | CATGGAAGTGTCGCCTGACAG |
| 40 | *Wnt4* | AGACGTGCGAGAAACTCAAAG | GGAACTGGTATTGGCACTCCT |
| 41 | *Axin2* | TGACTCTCCTTCCAGATCCCA | TGCCCACACTAGGCTGACA |
| 42 | *Gapdh* | AGGTCGGTGTGAACGGATTTG | TGTAGACCATGTAGTTGAGGTCA |
